# Electromagnetic Medical Sensing

**DOI:** 10.3390/s19071662

**Published:** 2019-04-08

**Authors:** Amin Abbosh

**Affiliations:** School of ITEE, The University of Queensland, Brisbane, QLD 4072, Australia; a.abbosh@uq.edu.au

In recent years, electromagnetic (EM) techniques have been widely investigated and researched for different medical applications, from early diagnosis to therapy and monitoring. It is well known that biological tissues have specific dielectric properties, which are usually frequency dispersive. Any change in the physiological and pathological conditions of those tissues change those dielectric properties, and thus the response of the affected tissues to EM waves will change. Moreover, EM waves interact strongly with biological tissues, and thus, they deliver via that interaction specific energy to those tissues. Consequently, electromagnetic sensors can be designed and used to detect, image, and monitor those changes or as a therapeutic tool for the affected tissues. Moreover, any research on medical sensing based on EM techniques will need to look at dosimetry levels and ensure subject safety.

Many techniques have been formulated to enable the use of electromagnetic sensing methods in either non-invasive or invasive assessment, therapy and monitoring of the functional and pathological conditions of tissues. The investigated techniques and relevant devices and systems extend across many topics, such as biosensors, electromagnetic imaging, digestible sensors, electromagnetic capsule, electromagnetic-guided therapy, hyperthermia or hypoglycaemia sensors, MRI compatibility of sensors, implantable sensors, transcranial magnetic stimulation, tomography, microwave ablation, wearable sensors, etc.

This Special Issue of *Sensors* aims at reporting on some of the recent research efforts on this increasingly important topic. The six accepted papers in this issue cover medical imaging, sensing and monitoring using different techniques and sensors, such as resonance-based reflector antennas with fast frequency domain processing [1], wideband microwave tomography [2], transmission-based open-ended coaxial dielectric probes [3], bio-impedance spectroscopy [4], magneto-elastic sensors [5], and electromagnetic tracking for surgical navigation and medical robotics [6]. This Special Issue also includes three reviews. Two of them discuss glucose sensing and monitoring: non-invasive electromagnetic sensing of glucose [7], and invasive to minimally-invasive to non-invasive techniques, devices and sensors for glucose monitoring [8], whereas the third review investigates electromagnetic–acoustic biomedical sensing [9].

In [1], a three-dimensional electromagnetic torso scanner system is presented. The authors of the system, which operates at the low microwave frequency band, aim at providing a complimentary imaging modality to supplement conventional systems for pathologies in the chest and upper abdomen such as pulmonary abscess, fatty liver disease and renal cancer. The system includes an array of resonance-based reflector antennas, which are installed on a movable flange to mechanically scan the human chest and upper abdomen with an accuracy of about 1 mm. To process the collected data, a fast frequency-domain imaging method in conjunction with a slice interpolation technique is used to generate three-dimensional images. To validate the system, experiments were performed on a torso phantom that emulates pulmonary abscess. The authors of [1] concluded that their results indicate the feasibility of the system, but future clinical tests are needed to confirm its reliability.

The use of wideband microwave tomography system for medical application is discussed in [2]. The paper includes the design of a prototype system, which operates across the frequency band 1–3 GHz and uses printed monopole antennas that are immersed in a coupling liquid. The paper discusses the imaging setup, signal transmission level and antenna sensitivity to different targets and coupling liquids. The imaging performance of the system is evaluated using a modified two-dimensional distorted Born iterative algorithm, on simple cylindrical targets. The authors of [2] inferred based on the results the potential of the technique in biomedical applications.

In [3], a transmission-based, open-ended coaxial dielectric probe is proposed for potential use in clinical situations. The probe aims at addressing limitations of typical reflection-based dielectric probes. The developed probe uses a low profile, open-ended coaxial cable that enables characterizing in compact spaces. One of the main features of the proposed probe is its sensing depth, which can be extended significantly compared to conventional probes. By verifying the performance on several homogeneous liquids with different dielectric properties, the initial results indicate a reasonable agreement between the transmission-based probe and commercial, reflection-based probes. The authors of [3] concluded based on the included results that the proposed probe is less affected by poor sample contact or cable bending, opening the door for its potential clinical applications.

The authors in [4] explained that oral diseases, which have the potential for malignant transformations, cause abnormal structural changes that are clinically assessed by visual inspection and palpation. To improve accuracy, this paper investigates using non-invasive bio-impedance spectroscopy as a possible option to improve the diagnostics of malignant lesions by conducting experiments on ex vivo pork oral tissues. The included results show that the studied tissues have unique spectra accompanied by significant differences in both impedance magnitude and phase. The authors of [4] concluded that bio-impedance spectroscopy deserves further investigation to clarify its potential to detect some specific pathological tissue changes.

In [5], a magneto-elastic sensor is proposed as an in-vitro wireless and passive method to monitor and assess the degradation rate of magnesium-based artificial bone, which can be used as an implant to repair a bone. The sensor is embedded in the neutral surface of the artificial bone by an adhesive. A modified simulated body fluid mimicking the human internal environment, and another media to accelerate degradation are used to immerse the artificial bone in the test period. The artificial bone is then tested daily on a self-developed experimental platform to monitor the relative output power under external forces. The authors explain that their obtained results show feasibility of the technique as the output power of the sensing coil gradually increases with bone degradation.

The authors of [6] discuss the role of electromagnetic tracking in surgical navigation and medical robotics as a positional and orientation reference. The authors noted that measurement errors caused by magnetic distortions is one of the technology’s principal shortcomings. This paper describes a method of detecting static magnetic distortions using only the magnetic field transmitting array. To that end, an available transmitter is modified to enable simultaneous transmission and reception of the generated magnetic field. A mutual inductance model is developed so that deviations from control measurements indicate location, magnitude and material of the field distortion. The authors concluded that their work enables optimizing the placement of magnetic transmitters by characterizing a distorter’s effect within the tracking volume without the need for additional hardware.

In [7], a review on non-invasive electromagnetic sensing of glucose is presented. The paper explains that diabetic patients need long-term and frequent glucose monitoring to assist in insulin intake. In this review, several electromagnetic technologies for non-invasive glucose measurement including infrared spectroscopy, photoacoustic spectroscopy, Raman spectroscopy, fluorescence, optical coherence tomography, Terahertz spectroscopy, and microwave sensing are discussed. The fundamentals of each method, its system setup, and experimental results are explained. The authors of [7] concluded that despite the promising achievements, no established product has obtained regulatory approval or survived a marketing test so far. To that end, the paper discusses the limitations and prospects of these techniques.

The authors of [8] explain that current glucose monitoring methods for the ever-increasing number of diabetic people are invasive, painful, time-consuming, and an ongoing financial burden. Thus, non-invasive glucose monitoring has been researched with the aim to overcome these limitations. This review offers a comprehensive up-to-date report on the leading technologies for non-invasive and minimally-invasive glucose monitoring sensors, devices currently available in the market. It also discusses the regulatory framework for performance assessment, newly proposed approaches, and relevant processing and prediction algorithms. The review also discusses the future trend of glucose detection by analyzing the usage of the different bands in the electromagnetic spectrum. The review painted a positive view on the potential of non-invasive and minimally-invasive glucose monitoring. The authors concluded that the adoption and use of new technologies for glucose detection is unavoidable and closer to become a reality.

The paper [9] reviews the theories and applications of different electromagnetic-acoustic techniques. The investigated techniques include light-induced, microwave-induced, thermo-acoustic, magnetic-modulated thermo-acoustic, and X-ray-induced thermo-acoustic methods. The review covers excitation of the fields using laser, microwave, magnetic field, and X-ray, and the generated acoustic waves. The explained applications include structural imaging, blood flowmetry, thermometry, dosimetry for radiation therapy, hemoglobin oxygen sensing, fingerprint imaging, glucose sensing, and pH sensing. The authors concluded that electromagnetic-acoustic techniques have potential in both pre-clinical research and medical practice.

In summary, there is a huge potential for electromagnetic techniques in medical detection, monitoring and sensing. However, there are still many challenges to address before those techniques can become a reliable clinical reality. A comprehensive review of the literature shows that many researchers in the past underestimated those challenges. They were highly optimistic based solely on oversimplified modelling, simulations or lab tests, only to be disappointed later by the outcome of their clinical results. Young researchers entering the field should be cautioned against generalizing their simplified simulations or lab environments to the clinical environment. They should not rush to draw strong conclusions based solely on simplified modelling. Testing on a static model in the lab or a model in the computing facility is far different from testing in the clinical environment: Those modelling, simulations and lab tests are under the full control of the researcher; parameters are known and assumptions are possible, whereas the clinical environment has a lot of uncertainties and uncontrollable factors. The potential of electromagnetic medical sensing is great, but the challenges are great too. But, we all know that great challenges generate great, rewarding, and impactful research—without challenges, research is pale and pointless.
